# A geographically matched control population efficiently limits the number of candidate disease-causing variants in an unbiased whole-genome analysis

**DOI:** 10.1371/journal.pone.0213350

**Published:** 2019-03-27

**Authors:** Matilda Rentoft, Daniel Svensson, Andreas Sjödin, Pall I. Olason, Olle Sjöström, Carin Nylander, Pia Osterman, Rickard Sjögren, Sergiu Netotea, Carl Wibom, Kristina Cederquist, Andrei Chabes, Johan Trygg, Beatrice S. Melin, Erik Johansson

**Affiliations:** 1 Department of Medical Biochemistry and Biophysics, Umeå University, SE Umeå, Sweden; 2 Computational Life Science Cluster, Department of Chemistry, Umeå University, SE Umeå, Sweden; 3 Division of CBRN Security and Defence, FOI–Swedish Defence Research Agency, SE Umeå, Sweden; 4 Science for Life Laboratory, Department of Cell and Molecular Biology, Uppsala University, SE Uppsala, Sweden; 5 Department of Radiation Sciences, Oncology, Umeå University, SE Umeå, Sweden; 6 Unit of research, education and development, Region Jämtland Härjedalen, SE Östersund, Sweden; 7 Science for Life Laboratory, Department of Biology and Biological Engineering, Chalmers University of Technology, SE Göteborg, Sweden; 8 Department of Medical Biosciences, Medical and Clinical Genetics, Umeå University, SE Umeå, Sweden; Oslo Universitetssykehus, NORWAY

## Abstract

Whole-genome sequencing is a promising approach for human autosomal dominant disease studies. However, the vast number of genetic variants observed by this method constitutes a challenge when trying to identify the causal variants. This is often handled by restricting disease studies to the most damaging variants, e.g. those found in coding regions, and overlooking the remaining genetic variation. Such a biased approach explains in part why the genetic causes of many families with dominantly inherited diseases, in spite of being included in whole-genome sequencing studies, are left unsolved today. Here we explore the use of a geographically matched control population to minimize the number of candidate disease-causing variants without excluding variants based on assumptions on genomic position or functional predictions. To exemplify the benefit of the geographically matched control population we apply a typical disease variant filtering strategy in a family with an autosomal dominant form of colorectal cancer. With the use of the geographically matched control population we end up with 26 candidate variants genome wide. This is in contrast to the tens of thousands of candidates left when only making use of available public variant datasets. The effect of the local control population is dual, it (1) reduces the total number of candidate variants shared between affected individuals, and more importantly (2) increases the rate by which the number of candidate variants are reduced as additional affected family members are included in the filtering strategy. We demonstrate that the application of a geographically matched control population effectively limits the number of candidate disease-causing variants and may provide the means by which variants suitable for functional studies are identified genome wide.

## Introduction

With the introduction of next-generation sequencing technologies, expectations were high that disease-causing genetic variants in familial diseases could be identified. However, the discoveries in recent years have in many cases been limited to the “low hanging fruit”, resolving familial diseases with a strong phenotype and an early age of onset and predominantly finding variants in coding regions and in known disease genes [1, 2]. A major reason for this is the high diversity of the human genome that leads to a large number of candidate disease-causing variants within any given family [3]. Prioritizing candidate variants for functional validation is difficult, often becomes biased towards already-known disease pathways, and has lately been shown to be of limited use in clinical settings [4, 5]. Large-scale functional studies are not possible to perform due to high cost and time considerations, thus limiting functional investigations to the strongest candidates.

Large-scale projects that attempt to map the functional elements of the genome, such as ENCODE, have suggested that as much as 80% of the genome might be involved in biological processes or interact with proteins [6]. Others have reported that 8% of the genome is evolutionarily constrained, arguing that these regions are functional, in contrast to the 1% representing regions coding for proteins [7]. Although there is a large span in the estimates of how much of the genome might be functional, such estimates imply that many disease-related variants are overlooked when restricting the analysis to the coding genome. Thus, it is of utmost importance to carry out unbiased whole-genome analyses. For this to be possible, the number of candidate disease-causing variants needs to be limited, preferably to a level where all remaining candidates can be assessed in functional assays.

**Fig 1:**
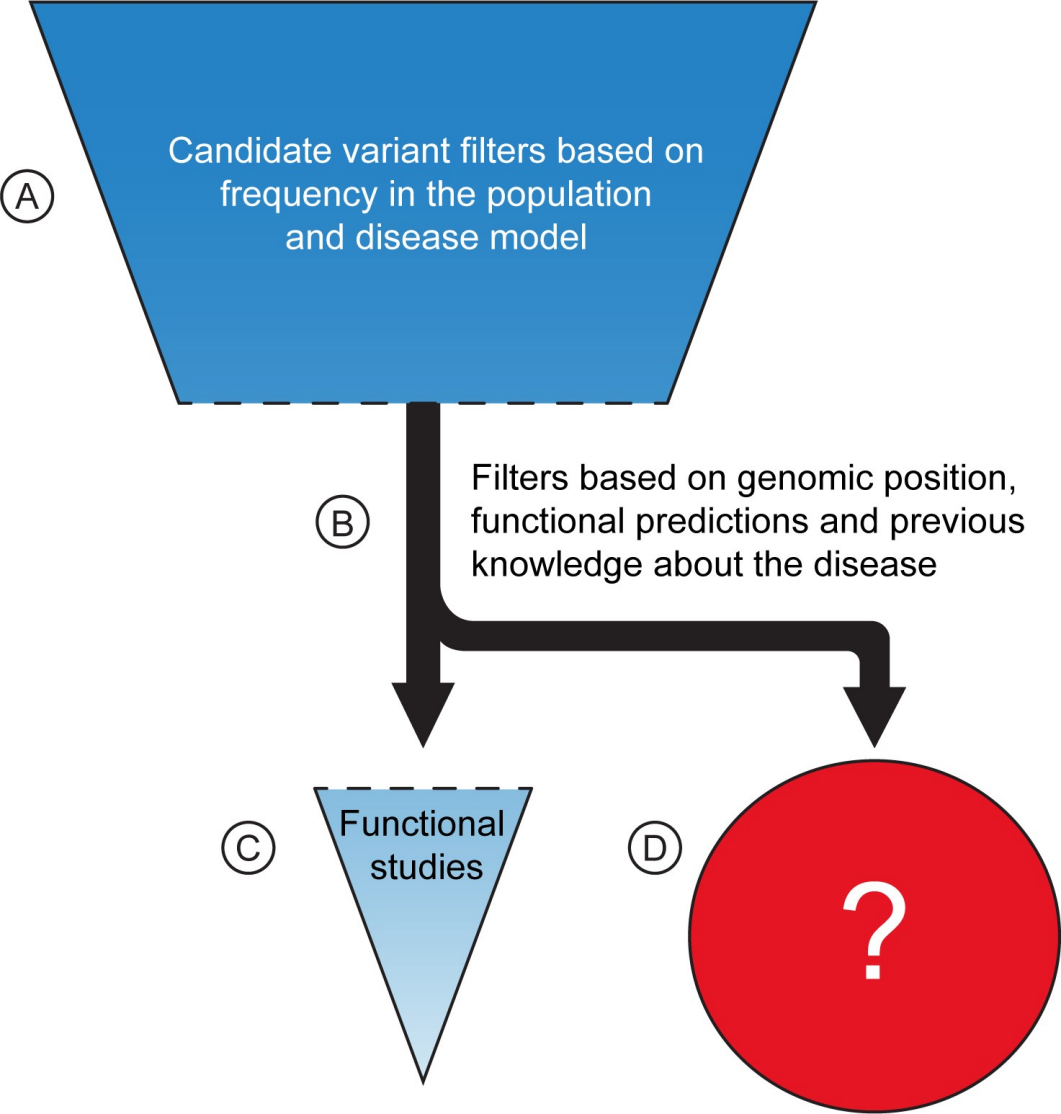
General filtering strategy to identify candidate disease variants. The scheme outlines a typical candidate disease variant filtering strategy whereby successive filtering steps are applied to reduce the number of candidate variants. (A) The initial steps removing variants highly unlikely to be disease causative. (B) The more stringent and error prone steps often applied to restrict the number of candidates for (C) functional analyses. The red circle (D) illustrates the bulk of variants removed in step (B). Step B should be avoided as there is a large risk that disease causing variants are discarded (D). This can in theory be achieved by improving the power of any of the steps in (A) e.g. by increasing family size (to reduce number of shared candidate variants among affected individuals) or by better knowledge about the genetic variation in the geographic area where the family has its origins (to exclude variants with a local MAF incompatible with the prevalence of the disease).

Disease-variant filtering strategies today include initial steps that remove variants that are highly unlikely to be disease causing e.g. based on their frequency in the general population or because they do not segregate according to the disease model (Fig 1A). This is typically followed by filtering steps based on previous knowledge and functional predictions (Fig 1B), that can be highly constraining and are prone to accidental discarding of true disease causing-variants and therefore needs to be used with caution [8]. Publically available population studies are valuable for removing common variants that are not causative of rare hereditary diseases. These datasets, however, typically lack phenotypic information such as age and disease state, making decisions on suitable minor allele frequency (MAF) cut-offs difficult. Further, and more importantly, they do not cover local variation [3]. Here we have whole genome sequenced (WGS) a control population from the same geographical area as a sequenced family carrying an autosomal dominant form of colorectal cancer. We demonstrate that the use of a geographically matched control population efficiently eliminates the genetic variation that is not associated with disease, reducing the number of candidate variants to a level where manual curation and functional studies are a viable option–without the use of previous knowledge or functional predictions. This enables true whole genome analysis of families with autosomal dominant diseases.

## Results

### Successively increasing the number of sequenced affected family members does not allow an unbiased identification of disease-causing variants

**Fig 2:**
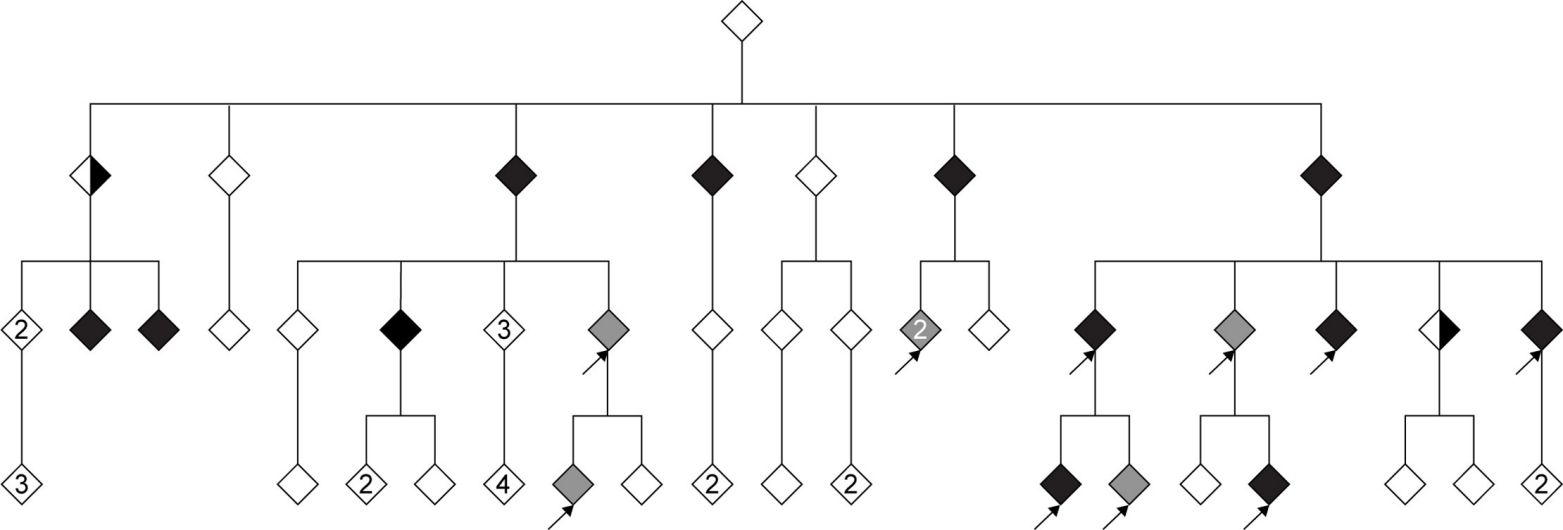
Pedigree of family CRC1. The pedigree is abbreviated to increase readability and to protect the family from identification. Half-filled diamonds represent individuals with stomach cancer, filled black diamonds represent individuals with colorectal cancer, grey diamonds represent individuals who have had four or more adenomas removed, and empty diamonds represent individuals with undetermined phenotype. In case a diamond represents multiple individuals, the number is given within the symbol. Whole genome sequenced affected individuals are indicated with an arrow.

**Fig 3:**
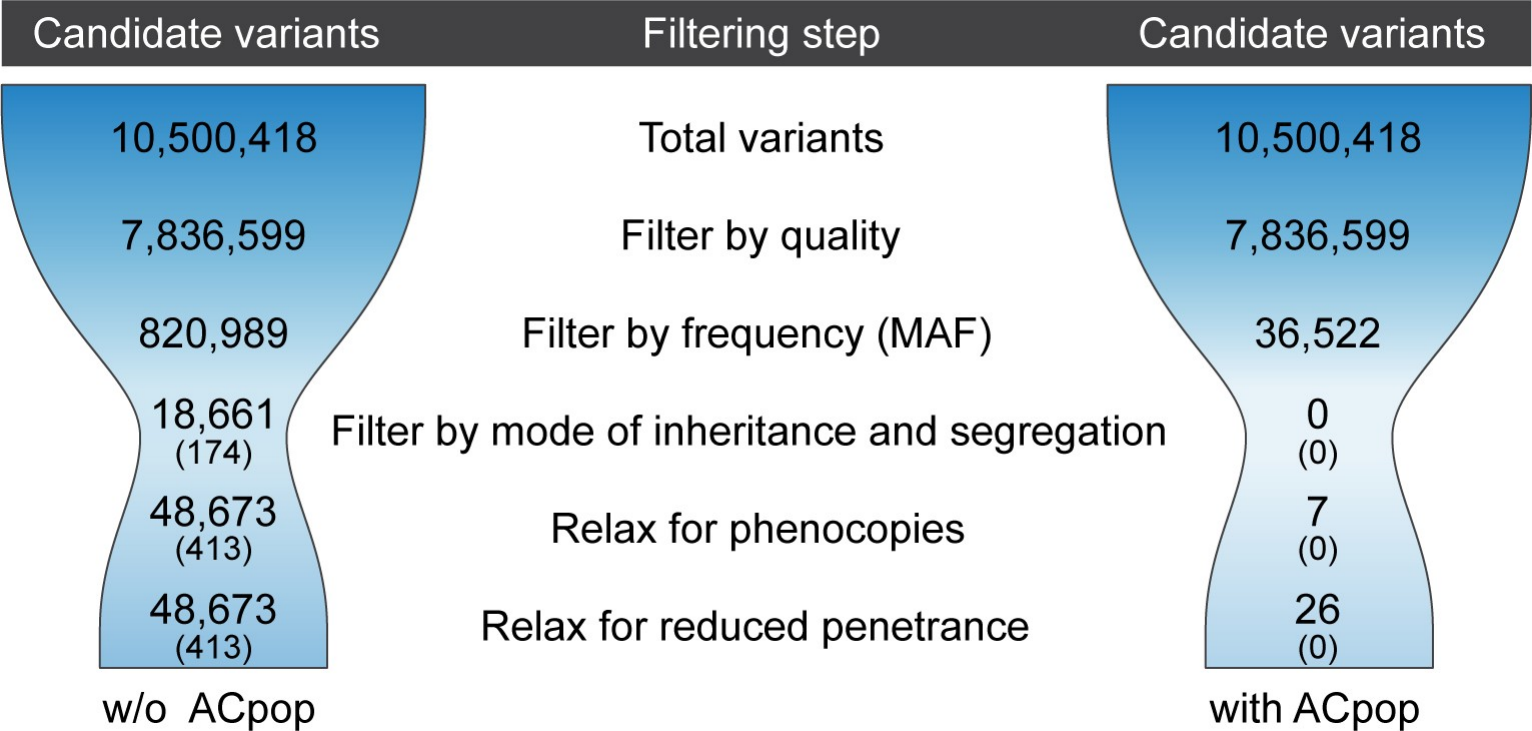
Number of candidate variants at successive steps of filtering and relaxation, with and without ACpop. The numbers represent candidate variants in family CRC1 with and without access to the control population ACpop. The first step of the filtering strategy consisted of removing variants of poor quality. Thereafter, variants that were hypothesized to be non-disease-causing were removed. A minor allele frequency cut-off (MAF>1%) was used for the six public variant databases while all variants found in ACpop were removed. In the next step, variants were conditioned to exist in the 11 affected individuals. Two additional steps were applied where the conditions were relaxed. The first was to allow for two sporadic cases of colorectal cancer among the 11 affected individuals, and the second was to allow for reduced penetrance of the disease-causing variant in ACpop.

**Fig 4:**
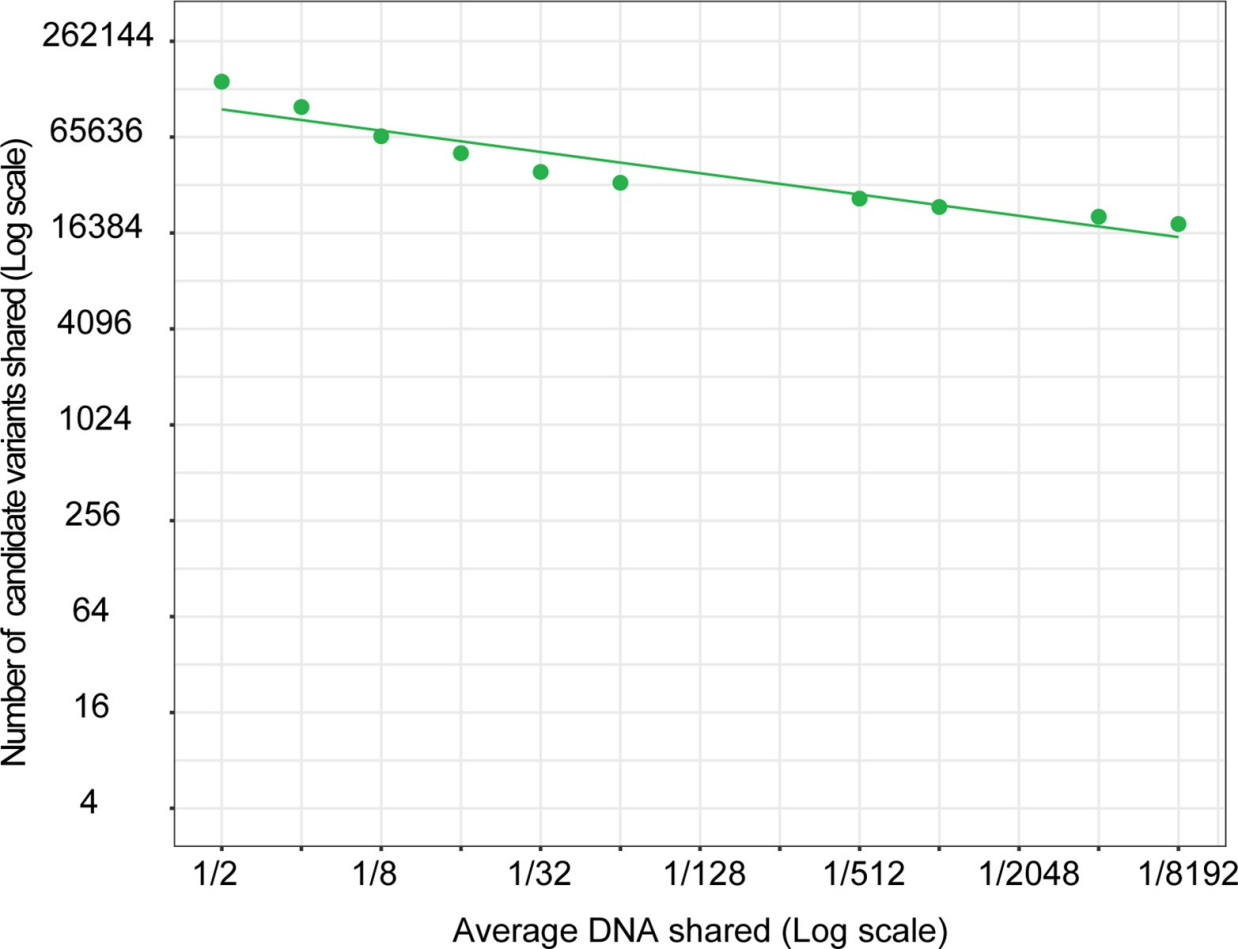
Number of shared candidate variants versus degree of relatedness. Plot over number of shared candidate disease-causing variants when successively adding up to 11 affected individuals from family CRC1 to the filtering analysis. The average reduction of the number of candidate variants for each halving of the fraction of the genome, expected to be shared by the affected individuals, is 20%.

It is well known that whole-genome sequencing (WGS) the human genome results in a daunting number of genetic variants, forcing many disease studies to restrict their analysis to the coding genome (the exomes) or even to nonsense mutations (Fig 1). In an attempt to avoid this, we used genealogical studies to piece together a large family with an autosomal dominant form of colorectal cancer (family CRC1) (Fig 2). The family consists of over 100 known family members out of which 20 are classified as affected (diagnosed with cancer or >4 adenomas). Blood samples were available for 11 of the affected individuals, and they were all sent for whole-genome sequencing. We applied a filtering strategy where we first removed variants of poor quality and variants common (MAF >1%) in any of six publically available variant datasets (1000g, ESP, ExAC, UK10K, GoNL, and deCODE [3, 9–12]).Thereafter we required that all affected individuals share a variant for it to be considered a candidate (Fig 3). Increasing the number of affected family members in the filtering analysis is expected to lead to a reduction in the number of candidate disease-causing variants. This follows from that the fraction of the genome that is shared between all affected individuals will be reduced as more individuals are added. As expected, we saw that progressively adding affected individuals in the filtering analysis gradually reduced the number of shared candidate disease-causing variants, but only by 20% on average each time the shared fraction of the genome was halved (Fig 4). The available 11 affected individuals shared as little as 1/8,192 of their genome, but still 18,661 candidate variants across the genome remained for functional analysis (Fig 3). Removing all variants found in the six databases (a cutoff of MAF>0%) gave little effect as 18,329 variants still remained. For a smaller study including 3–5 affected individuals that share 1/16–1/64 of their genome, there would be an overwhelming 33,000–52,000 candidate variants left genome wide (Fig 4).

The large number of candidate variants remaining after the filtering analysis illustrates that we have a substantial pool of variants in northern Sweden that are rare or absent in other populations, i.e. they were not excluded when applying MAF filters to remove variants that are common in public variant datasets (Fig 3). Additionally, because the number of candidate variants did not decrease at the expected rate when increasing the number of sequenced affected individuals (Fig 4), we hypothesized that some of these variants are common in the local population and for that reason not linked to hereditary colorectal cancer. This suggested to us that sequencing a geographically matched control population could be an effective approach to reduce the number of candidate disease-causing variants to a manageable level.

### Sequencing of a geographically matched control population (ACpop) to describe the local genetic variation

**Fig 5:**
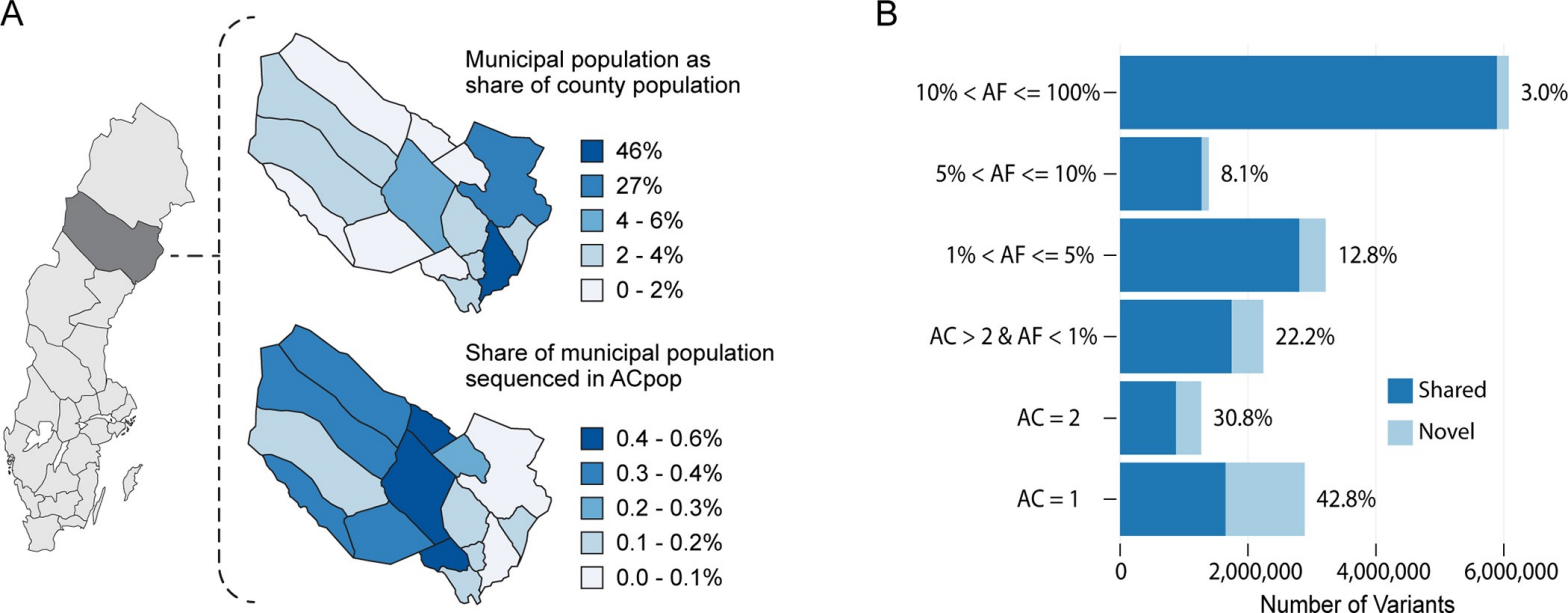
Representation of ACpop in the total population of Västerbotten County and the allele frequency distribution. (A, left) The location of Västerbotten County in Sweden. (A, top) The municipal share of the Västerbotten County population. (A, bottom) The share of the municipal population that has been sequenced in ACpop. The maps were modified from an open map of the Västerbotten County and Sweden, published by the Swedish Mapping, Cadastral, and Land Registration Authority URL: https://www.lantmateriet.se/en/maps-and-geographic-information/oppna-data/hamta-oppna-geodata/ (B) The proportion of variants that are novel (light blue) in a comparison to the six public databases (1000g, ESP, ExAC, UK10K, GoNL, and deCODE) stratified by allele frequency. The fraction of variants shared by ACpop and at least one of the six public databases are found in dark blue. AF stands for allele frequency and AC for allele count.

Family CRC1 originates from Västerbotten, a county situated in northern Sweden with a population of approximately 265,000 people (Fig 5A). Our control population named ACpop was decided to consist of 300 individuals and samples were selected from the Västerbotten Intervention Programme (VIP) [13], a public health program for which all inhabitants of the county turning 40, 50, and 60 years old are invited. The inclusion criteria for the control population were that an individual should have reached an age of at least 80 years and have had no diagnosed cancer. Out of the approximately 95,000 individuals in the VIP 3,502 individuals met these criteria. In order to detect as much of the county’s genetic variation as possible, half of the 300 samples were spread evenly over the 15 municipalities within Västerbotten County while the other half was used to enforce municipalities of higher population density. In addition, to minimize selection bias and maximize diversity, samples within each municipality and gender were selected according to a systematic design where 27 phenotypic and lifestyle variables were taken into consideration (material and methods). This approach resulted in the selection of individuals representing 0.2–0.6% of the population in each inland municipality and 0.04–0.2% of the population in each municipality along the coast (Fig 5A). The lower coverage along the coast is due to that 78% of the population resides in coastal municipalities (Fig 5A).

A total of 17,070,830 variants–including 14,414,452 single nucleotide polymorphisms (SNPs) and 2,656,378 insertions/deletions (indels)–were found in ACpop. Among these, 2,828,043 variants were unique to ACpop compared to the six publically available variant datasets 1000g, ESP, ExAC, UK10K, GoNL, and deCODE. Out of these 2.8 million variants, 706,315 (25%) had an allele frequency of over 1% (Fig 5B). In other words, 4.1% of the total call set consisted of variants that are common in Västerbotten County but have not previously been seen in any of the other six studies.

### The local control population (ACpop) effectively limits the number of candidate disease associated variants in family CRC1

The impact of the matched control population became apparent when removing all ACpop variants from the variants in family CRC1. The 11 affected individuals together carried 7.8 million variants (Fig 3), and this was reduced by 10 fold (to 820,989 variants) when removing variants common (MAF >1%) in any of the six publically available variant datasets (Fig 3). Removing not only the common variants from these datasets but all variants (not recommended because of the lack of phenotypic matching within these datasets) only led to a small further reduction of number of variants to 643,818. Including ACpop in the analysis, however, reduced the number of variants another 20-fold to 36,522 (Fig 3). Excluding the 6 public datasets from the filtering only increased the number of candidate variants to 40,671, illustrating that we no longer need to depend on public datasets.

None of the 36,522 variants remaining in the above analysis were shared by all 11 affected individuals in family CRC1, thus 0 candidate disease-causing variants were left in the filtering analysis (Fig 3). However, because the lifetime risk of colorectal cancer is 4.5% [14], it is very likely that phenocopies (sporadic cases of colorectal cancer) are present in the pedigree of more than 100 individuals in family CRC1. In addition, the penetrance of the disease-causing variant cannot be assumed to be 100%, meaning that even though we made a careful selection of cancer free elderly individuals there is a risk that an individual in the control population carry the disease-causing variant. Relaxation of the filtering strategy allowing for one individual in ACpop to carry the disease causing variant and assuming at most 2 phenocopies in the family we end up with 26 candidate disease-causing variants genome wide (Table 1). Without access to ACpop this number would have been 48,673 candidates out of which 413 are exonic (Fig 3). None of the 26 candidates were exonic (Table 2). Allowing additional carriers in ACpop slowly increases the number of candidate disease variants. However, allowing for as many as seven individuals in ACpop to carry the variant (extremely unlikely) we still have below 100 candidate variants genome wide out of which none are exonic (Table 1). This illustrates that the 413 exonic variants remaining when ACpop is not included (Fig 3), and which are more likely to be predicted functional than non-coding variants, are unlikely to be the disease-causing variants because of their high frequency in the local population.

**Table 1:** Cumulative number of candidate variants depending on number of phenocopies and carriers in ACpop. Top right corner represents number of shared candidate variants by all 11 affected individuals but not present in ACpop. In each column we introduce the maximal number of carriers in ACpop. In each row we introduce the maximal number of sporadic cases in the family (number of phenocopies).

		Number of carriers in ACpop								
		0	1	2	3	4	5	6	7	8
**Number of phenocopies**	**0**	0	0	0	0	0	0	0	0	0
	**1**	0	3	3	3	3	3	3	4	4
	**2**	7	26	31	34	59	66	79	91	108
	**3**	85	166	246	339	491	608	708	806	939
	**4**	330	621	1039	1390	1831	2275	2589	2908	3231

**Table 2:** Summary of the 26 variants remaining after filtering analysis of family CRC1.

Position	Change (ref/alt)	Type	Gene (RefSeq)	dbSNP 151	1000 genomes EUR (aaf*)
chr3:77201736	G/A	Intron	ROBO2	rs62251809	0.006
chr9:68504750	G/T	intergenic	None	rs201332223	-
chr10:17301230	A/G	intergenic	None	-	-
chr20:1813341	G/A	intergenic	None	rs180703964	0.002
chr20:1869125	C/T	intergenic	None	rs146934631	0.003
chr20:1872351–1872354	ACCT/A	upstream gene	SIRPA	rs778710946	-
chr20:2111830	G/T	Intron	STK35	rs35061411	0.001
chr20:2493371	T/G	upstream gene	ZNF343	rs147087733	0.003
chr20:2690886	C/T	Intron	EBF4	rs190090026	0.001
chr20:2733490	C/T	Intron	EBF4	rs187805451	0.002
chr20:2738462	G/T	Intron	EBF4	rs151095258	0.009
chr20:2790980	C/T	upstream gene	C20orf141	rs952465216	-
chr20:2845312–2845321	ATGGGGCGTG/A	Intron	PTPRA	rs748038717	0.008
chr20:3922982–3922983	AT/A	Intron	RNF24	rs879326719	-
chr20:3998030	C/T	upstream gene	RNF24	rs889095036	-
chr20:4222335	T/C	Intron	ADRA1D	rs191072065	0
chr20:6815078–6815090	AAGAAAGAAAGAG/A	intergenic	None	rs144882419	-
chr20:7752380	C/A	intergenic	None	-	-
chr20:7887402	C/A	Intron	HAO1	-	-
chr20:8067390	T/C	intergenic	None	-	-
chr20:8126125	G/A	Intron	PLCB1	rs143541837	0.003
chr20:8228298	C/A	Intron	PLCB1	rs534663667	0.003
chr20:8248534	T/G	Intron	PLCB1	rs191574108	0.003
chr20:8302274	A/G	Intron	PLCB1	rs182705116	0.002
chr20:8482637	T/C	Intron	PLCB1	rs753952228	-
chr20:8541488	A/G	Intron	PLCB1	-	-

*Allele frequency of the alternative allele (non-reference)

**Fig 6:**
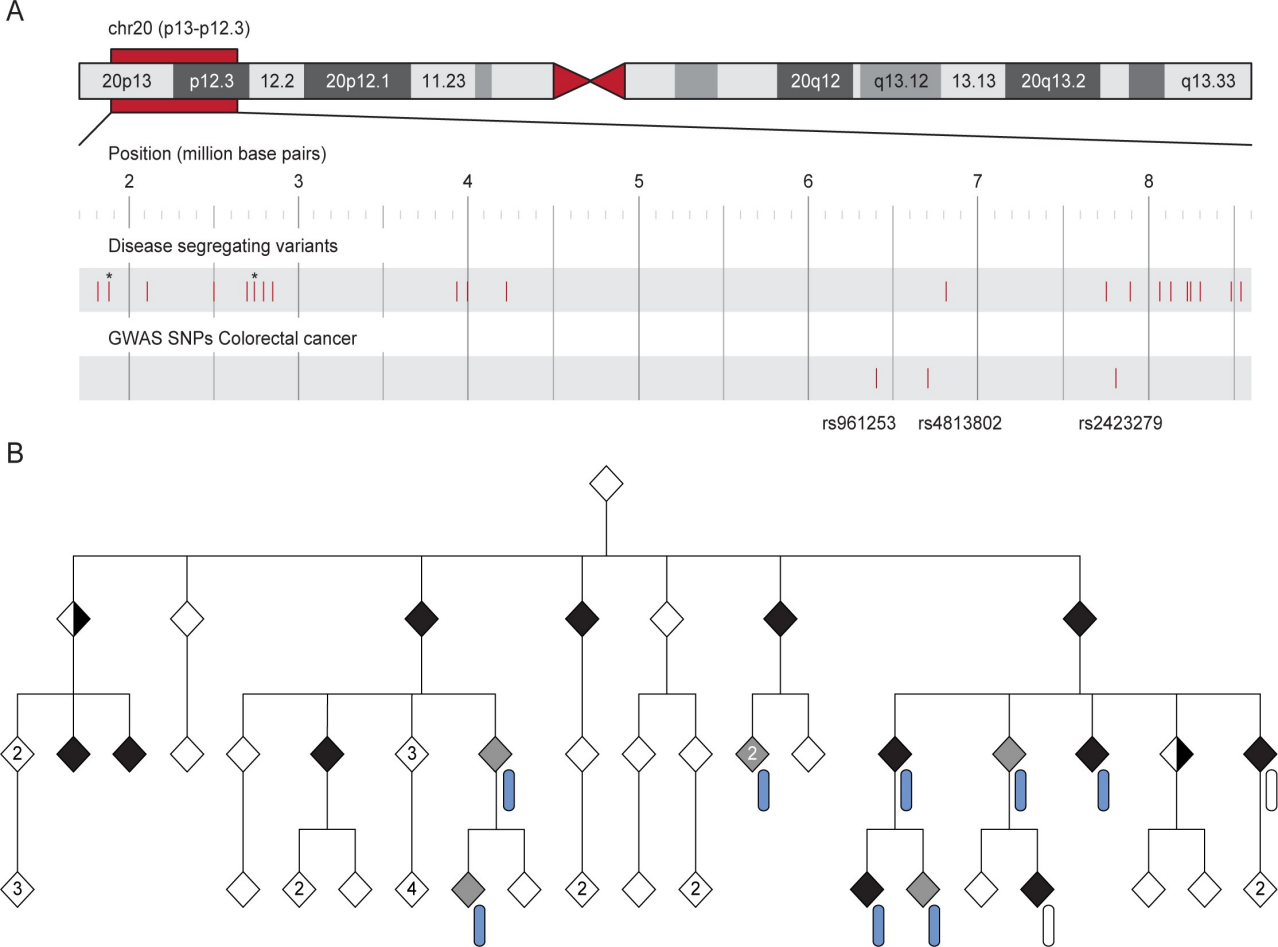
Visualization of the haplotype on chr20 and the segregation within family CRC1. (A) Figure adapted from the UCSC Genome Browser illustrating the positions of the 23 candidate variants on chr20. The relevant section is enlarged below the whole chromosome. The three SNPs found to be associated with colorectal cancer in previous GWAS are depicted in the track below. An asterisk represents two variants in close proximity. (B) An abbreviated pedigree of family CRC1 showing the segregation of the haplotype within the family. Filled black diamonds represent individuals with colorectal cancer, half-filled diamonds represent individuals with stomach cancer, grey diamonds represent individuals who have had four or more adenomas removed, and empty diamonds represent individuals with undetermined phenotype. The haplotype on chr20 is represented by a rounded rectangle. Blue color indicates a carrier of the haplotype and an empty symbol denotes individuals found not to carry the haplotype.

The 26 variants from the above analysis were considered for initial downstream evaluation (Table 2). Twenty-three of the 26 candidate variants reside on the same 7.4 Mb haplotype on chr20p13-p12.3 (Fig 6). The remaining three variants were found on chr3, chr9 and chr10. The haplotype on chr20 interestingly covers three previously identified risk SNPs for colorectal cancer rs2423279, rs961253 and rs4813802[15–18]. Five of the 26 variants were not found in the latest release of dbSNP (build 151) and were therefore considered novel. Two of these resided on opposite sides of one of the three GWAS risk SNPs (rs2423279) and around 1Mb downstream of the other two risk SNPs (rs961253 and rs4813802). Screening another 95 families originating from the same geographical region and with increased risk of colorectal cancer we identified individuals with colon cancer that carried these two variants in two other families. In summary, these data high-lights the 2 variants as of interest but functional studies are required before a disease-causing variant can be confirmed and a molecular mechanism can be proposed.

### ACpop enables a 50% reduction of candidate disease-causing variants when the genome shared by affected individuals are halved

To determine the impact of the control population when investigating smaller families and to see what effect a small shift in the family’s geographical origin might have on the number of candidate variants, we whole genome sequenced two unrelated trios, one from Västerbotten (TRIO1) and one from a neighboring county (TRIO2). In both cases, one parent and one child were affected, while the second parent was unaffected. The inclusion of ACpop in the filtering analysis resulted in 2,666 and 5,556 candidate variants in the two trios, respectively. This was a 28-fold decrease of candidate variants in the trio from Västerbotten as compared to only relying on the six public variant datasets and a 16-fold reduction in the trio from outside Västerbotten (Table 3). Out of the candidate variants in the two trios, 44 and 137 were exonic, respectively (Table 3). Variants that gave either a truncation or an amino acid substitution with a predicted effect on protein function were limited to 9 and 27, respectively. We conclude that it is still not sufficient to sequence a trio for identifying a variant causing an autosomal dominant disease unless the variant is deleterious for protein function. It is also clear that even a relatively small geographic distance between the studied family and the control population has a great impact on the final number of candidate variants.

**Table 3:** Candidate variants after filtering analysis in two trios.

	Without public datasets				With public datasets*			
Families	Without ACpop		With ACpop		Without ACpop		With ACpop	
	All	Exonic	All	Exonic	All	Exonic	All	Exonic
TRIO1	830,500	12,675	3,187	51	73,432	959	2,666	44
TRIO2	853,374	13,164	6,396	148	87,708	1190	5,556	137

*Dataset used for filtering: 1000 genomes, Exac, ESP, UK10K, deCODE, and GoNL

**Fig 7:**
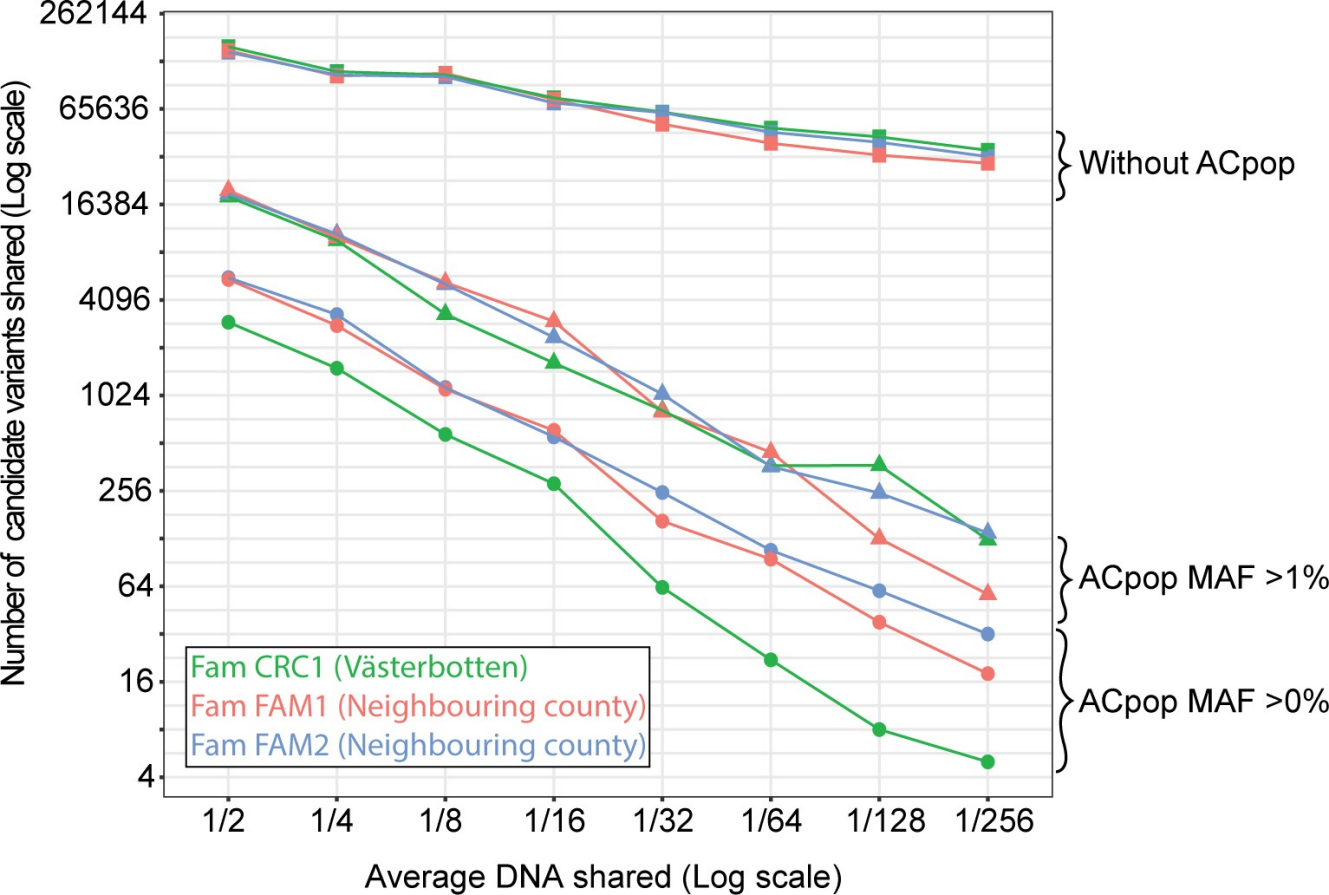
Number of shared candidate variants versus degree of relatedness. Individuals sharing 50% (e.g. two siblings) down to 0.4% of their genome were analyzed for number of shared variants. Three families were included, one from within Västerbotten County (family CRC1) and two from a neighboring county (families FAM1 and FAM2). The filtering analysis was performed three times with different applications of ACpop–(square) variants found in ACpop were not subtracted during the filtration, (triangle) all variants with an allele frequency over 1% in ACpop were subtracted during the filtration, and (circle) all variants present in ACpop were subtracted during the filtration. Variants with a MAF over 1% in six public variant databases (1000g, ESP, ExAC, UK10K, GoNL, and deCODE) were removed in all three filtering analyses.

As mentioned above, there is a relationship between the relatedness between sequenced individuals and the final number of candidate disease-causing variants. To further explore this relationship with access to ACpop, we carried out a series of filtrations on a decreasing level of relatedness in three larger families, one from Västerbotten (family CRC1) and two from a neighboring county (families FAM1 and FAM2). In this case, we did not include information about affection state, and we only considered the relatedness to exemplify how many candidate variants were shared. When ACpop was not included and only variants that are common (MAF >1%) in the six variant datasets were removed during the filtering analysis, we saw again on average a 20% decrease in the number of shared candidate variants when the fraction of shared genome was halved. When also including ACpop, we found on average a 50% decrease in the number of shared candidate variants when the fraction of shared genome was halved (Fig 7). This illustrates that only when variants common for the geographic region are removed from the analysis of a family is a filtration for a shared rare disease-causing variant that is not a “low hanging fruit” likely to be successful. Interestingly, only removing common variants (MAF >1%) in ACpop gave a comparable successive decrease (50%), but the total number of shared candidate variants was higher at each point (Fig 7) and the separation between the three families caused by geographical origin was lost.

## Discussion

In this study we have shown that it is possible to limit the final number of candidate disease-causing variants to a level where functional assays can be applied to identify the causal variant of a monogenic disease, even when using a whole-genome sequencing approach and without the use of common practice functional prediction filters that may produce false negatives. In other words, it is possible to successfully employ an unbiased filtering strategy where variants found across the entire genome, regardless of predicted functional importance, are included in the analysis. There is, however, an absolute requirement to remove variants that are common and therefore not disease-causing, in the geographic area from where the studied family originates.

We used a geographically matched control population to remove common variants in the selected geographic region. This could in theory also be achieved by sequencing a number of healthy individuals within the family. Acquiring these samples is, however, not trivial for many diseases. For example, in this study of hereditary colon cancer, the carrier status of unaffected family members is in many cases uncertain because they are younger than the average age of disease onset. Other challenges include reduced penetrance and preventive treatment that reduce the risk for disease. Parents who are married into the family should not be carriers but are often already deceased because of the late onset of the disease. In our case, we sequenced 27 family members in family CRC1 with uncertain phenotype (see Materials and methods). Including these individuals in the analysis improved the filtering results, but because of the uncertainty in the affection status were still too many candidate variants remaining. A rough estimate of the number of individuals with uncertain affection status that might carry the disease-causing variant is 9 (50% of the children of affected individuals and 25% of the grandchildren of affected individuals). Including these individuals in the filtering analysis and requiring that no more than 9 of the 27 individuals with unknown affection status can carry the variant gave a total of 1,347 candidate variants. This should be compared to the 26 candidate variants that remained when instead including ACpop in the filtering analysis. Thus, the contribution of variants found in family members with uncertain affection status was limited in the filtering strategy, while the carefully selected control population effectively removed non-disease-causing variants. Another benefit of using a control population is that it is not family specific and over time allows smaller families to be analyzed.

The explosive population growth over the last 400 generations has resulted in a large number of population specific rare variants [19]. Without knowledge about local genetic variation these variants that over time has become common in a specific geographic region while still rare in the global population, severely hinders the discrimination between disease variants and harmless local polymorphisms [3]. With a geographically matched control population these variants (globally rare but common in the local population) are identified. This explains why the 50% reduction of candidate variants when the genome shared by affected individuals is halved can be observed, and this is critical to reach a low number of candidate variants genome wide. This highlights the importance of being able to break down large population datasets into, for the analysis at hand, geographically meaningful subpopulations. The reason being that a geographically focused small population for MAF estimates provides a better estimate of common variants in the selected region, compared to a global estimate based on a million samples, (recently defined aims in both the US and Europe) where the local variation will be diluted. Performing filtration studies on a few related individuals in a region of interest, using available population frequencies, may provide an indication as to if a local control population must be established for filtering approaches.

Dopazo et al. recently illustrated the importance of a local Spanish control population in the filtering analysis of whole exome studies [20]. Here we broaden this to the whole genome, showing that with data on local MAF it is possible to reach very few candidate disease-causing variants also on a whole genome scale. In combination with the many large sequencing efforts worldwide today and the recent steep reduction in sequencing costs, this urges for a change of focus from exons to the entire genome, realizing the full potential of whole-genome sequencing.

## Methods

### Families included in the study

In this study, we have whole-genome sequenced blood samples from five families originating from northern Sweden (CRC1, TRIO1-2 and FAM1-2). Our primary focus was on family CRC1, a large family with high incidence of colorectal cancer. There are more than 100 known family members in CRC1, and there are medical records available for 60 of them (27 women and 33 men). Many of the individuals in the older generation were deceased, but blood samples could be obtained from 38 of the 60 family members with medical records (19 women and 19 men). These samples were all sent for whole genome sequencing. To illustrate the impact of the control population (ACpop) in our filtration strategy, we also collected blood and sequenced individuals from families called TRIO1-2 and FAM1-2. Blood samples have additionally been collected from at least one individual in another 95 smaller pedigrees. While these samples have not been sent for whole-genome sequencing, they were available for screening of candidate disease-causing variants.

The lineage of family CRC1 can be traced back to the second half of the 19th century, and the inheritance pattern in the pedigree suggests that the family suffers from an autosomal dominant form of hereditary colorectal cancer. The family comprises 12 cases of colon cancer and 2 cases of stomach cancer out of the 60 followed family members. The family has been offered surveillance with colonoscopies since the mid-1990s, and all suspected pre-stages of cancer (polyps and adenomas) are removed from the colon. This preventive treatment has decreased the number of tumors in younger generations [21–23]. A colorectal surgeon with extensive knowledge of colonoscopies therefore analyzed each family member’s colonoscopy results and identified another six affected individuals with a high number of adenomas (≥4) occurring over time at different locations. In total, 20 individuals in the family were defined as affected (8 women and 12 men), and 11 of these were whole genome sequenced (5 women and 6 men) (Fig 2). Sixteen of the 40 family members not classified as affected had between 1 and 3 adenomas of mainly small size removed from their colons. These individuals could not be defined as either sick or healthy because occasional adenomas are common in the population[24]. In addition, the average age of cancer diagnosis is 60 years, and many are older than 70 years before diagnosis. Many family members in the youngest generations were not yet 60 years. Taken together, we were unable to determine if unaffected individuals were healthy. In total, 11 affected family members and 27 family members with uncertain disease phenotype were whole genome sequenced. The study was approved by the ethics board in Umeå, Sweden (dnr 2012-151-31, dnr 2013-121-32, dnr 2015-489-32, and dnr 2017-370-32), and written informed consent was obtained from all living participants. The experiments were performed in accordance with relevant guidelines and regulations.

### Selection of the control population

In total, 300 people were sequenced for the control population, which was given the name ACpop (AC is the official county letter for Västerbotten County in Sweden). The samples were selected from the Västerbotten Intervention Programme (VIP)[13, 25], a cohort within the Northern Sweden Health and Disease Study (NSHDS), after ethical approval by the ethics board in Umeå, Sweden (dnr 2014-290-31). The VIP is a public health program in Västerbotten County, which is located in the northern region of Sweden and is the county from which family CRC1 originates. Within VIP, all inhabitants of Västerbotten County turning 40, 50, and 60 years old are invited to undergo a health checkup and, in addition, complete a questionnaire regarding their general health and lifestyle. Blood samples from the participants are stored for research purposes. To be considered for inclusion in ACpop, an individual must have donated a blood sample for research, reached an age of at least 80 years, and not been diagnosed with cancer. Out of approximately 95,000 individuals in the cohort, 3,502 met these inclusion criteria (2,079 women and 1,423 men). To map as many variants as possible from this geographic area, it was important to include samples from across Västerbotten County. As a baseline, half of the samples to be sequenced were spread evenly over the 15 municipalities of the county. The remaining half were dedicated to increase the frequency of sequenced individuals in the three most highly populated municipalities (Lycksele, Skellefteå, and Umeå), as well as municipalities that border neighboring counties. Of the 300 selected individuals, 150 were women and 150 were men. To maximize the diversity among selected individuals and to minimize selection bias, 27 phenotypic, health, and lifestyle-related variables were extracted from the VIP (S1 Table), and a principal component (PC) model was used to select the individuals to be sequenced from each municipality. The PC selection process was done separately for each gender and municipality. The 150 samples that form the baseline selection were selected from each model according to a full factorial design in two levels with one center point (S1A Fig). For the reinforced municipalities, the designs were extended with another full factorial design around each corner in the baseline design, as well as another four center points (S1B Fig).

### Whole-genome sequencing of blood samples

DNA was either extracted from blood samples provided by family members (FlexiGene-kitet, Qiagen Gmbh, Hamburg, Germany) or, in cases where there was a sample available, collected from the clinic. DNA samples from the 300 individuals in the control population were obtained from the NSHDS. All DNA samples underwent a quality check by the sequencing facility. DNA samples from families were collected and sequenced over a period of several years. Initially the samples were sequenced at Illumina (San Diego) and later at the Genomics Production site in Stockholm (NGI-S) and the SNP&SEQ facility in Uppsala (NGI-U) (Sweden) using the HiSeq2000 and HiSeqX systems. The 300 samples of the control population were sequenced at NGI-U using Illumina HiSeqX. PCR-free library preparation kits were used on all occasions, and samples were paired-end sequenced with a read length increasing from 100 bp to 150 bp. All samples were sequenced to at least 30× depth.

### Read alignment and data quality

The raw data from all samples were jointly processed to ensure that the same steps and software versions were used. All analyses were performed according to the GATK best practice[26, 27]. Briefly, reads were aligned to the 1000g fasta reference (b37) using BWA (v0.7.10-r789)[28]. Sorting, indexing, and marking of duplicates was done using Picard (v.1.118)[29], and realignment around indels was done using GATK (v3.3.0)[30]. Qualimap (v2.0.2) [31] was used to assure sample quality and to identify any deviating samples.

### Variant detection and quality filters

SNPs and small indels were called using HaplotypeCaller according to GATK’s best practice with version 3.3.0 of the GATK software suite [26, 27]. Briefly, all samples were called separately to produce one gVCF file per sample. The initial calls from families and the control population (gVCF files) were used to jointly generate one single VCF file containing all samples. This improves detection of variants and outputs data in a suitable format for downstream analysis. VQSR was used for quality filtering according to GATK recommendations. For SNPs, the truth sensitivity cutoff was set to 99.7 where the Ti/Tv ratio was equal to 2.1. For indels, which are called with a lower reliability, the truth sensitivity was set to 99.0. The allele frequencies of ACpop are published as a dataset that is retrievable from doi:10.17044/NBIS/G000005 (https://doi.org/10.17044/nbis/g000005), and hosted at the SweFreq searchable web-portal for specific allele-frequencies and read depth (https://swefreq.nbis.se).

### Relationship analysis

The software PLINK [32] was used to analyze relationships between individuals. This was used as a quality control against sample swap in the investigated families and to detect any cryptic relationships in ACpop. The—*genome* command in PLINK (v1.90b3) was used to calculate identity by descent (IBD) sharing between all samples. Variants that were included in the analysis were common (MAF >10%) biallelic SNPs with a successful genotyping rate of at least 99%. The variant set was LD-pruned using the—*indep-pairwise* command in PLINK with a window of 10,000 SNPs, a window step of 10 SNPs, and the r2 threshold set to 0.3. In total, 178,498 variants were used for the IBD calculation. Allele frequencies were calculated from ACpop. All samples from family CRC1 were predicted to have relationships in accordance with the pedigree. We found two pairs of siblings in ACpop. Additionally, a third-degree relationship was predicted between one individual in ACpop and one individual in family CRC1. The individual in ACpop was, however, not related to any of the other cousins of the CRC1 individual and was therefore assumed to be related on the mother’s side, who married into the family.

### Disease variant filtering

The VCF files from SNP and indel calling were decomposed and normalized using vt (v.0.5)[33], annotated using VEP (v. 82) [34], and loaded into Gemini (v.0.18.2)[35], a database framework for variant annotation and filtering. Gemini was used to find variants that were common to the affected individuals and rare in the public databases 1000g, ExAC, and ESP as well as in our control population. Different filtering setups (MAF cut-offs and phenocopy rate) were used as described in the main text. Bcftools (v1.3.1) [36] was used to remove variants from three additional public variant databases not present in Gemini (UK10K, deCODE, and GoNL).

### Variant phasing

Phasing of variants to obtain haplotypes within family CRC1 was done using SHAPEIT2 [37]. Samples from the family and the control population were included in the analysis. All biallelic SNPs with a genotyping rate above 1% were included. SHAPEIT2 was run with the *duohmm* option active. The number of conditioning states was set to 1000, the window size was set to 300, and the MCMC iterations were set to have 15 burn-in and pruning iterations and 50 main iterations.

### Validation of variant calls by Sanger sequencing

SNP regions were PCR amplified, and the DNA was sent to Eurofins Genomics for purification and sequencing (Plate Seq Kit PCR or Mix2Seq, Eurofins Genomics).

## Supporting information

S1 Table The variables used in the selection of ACpop. (XLSX)

S1 Fig Designs used for selection of samples for ACpop. The figure illustrates a schematic score plot of the first and second component from a PCA model. Pink dots represent the data points (samples) and diamonds indicate selected samples. The baseline selection for all municipalities was made according to a full factorial design (A), whereas for the more populous municipalities an extended design was used (B).
(TIF)
