# Supplementary figures and images for: A geographically matched control population efficiently limits the number of candidate disease-causing variants in an unbiased whole-genome analysis
Source: PLoS One. 2019 Mar 27;14(3):e0213350. doi: 10.1371/journal.pone.0213350 (PMC6436687; doi:10.1371/journal.pone.0213350)

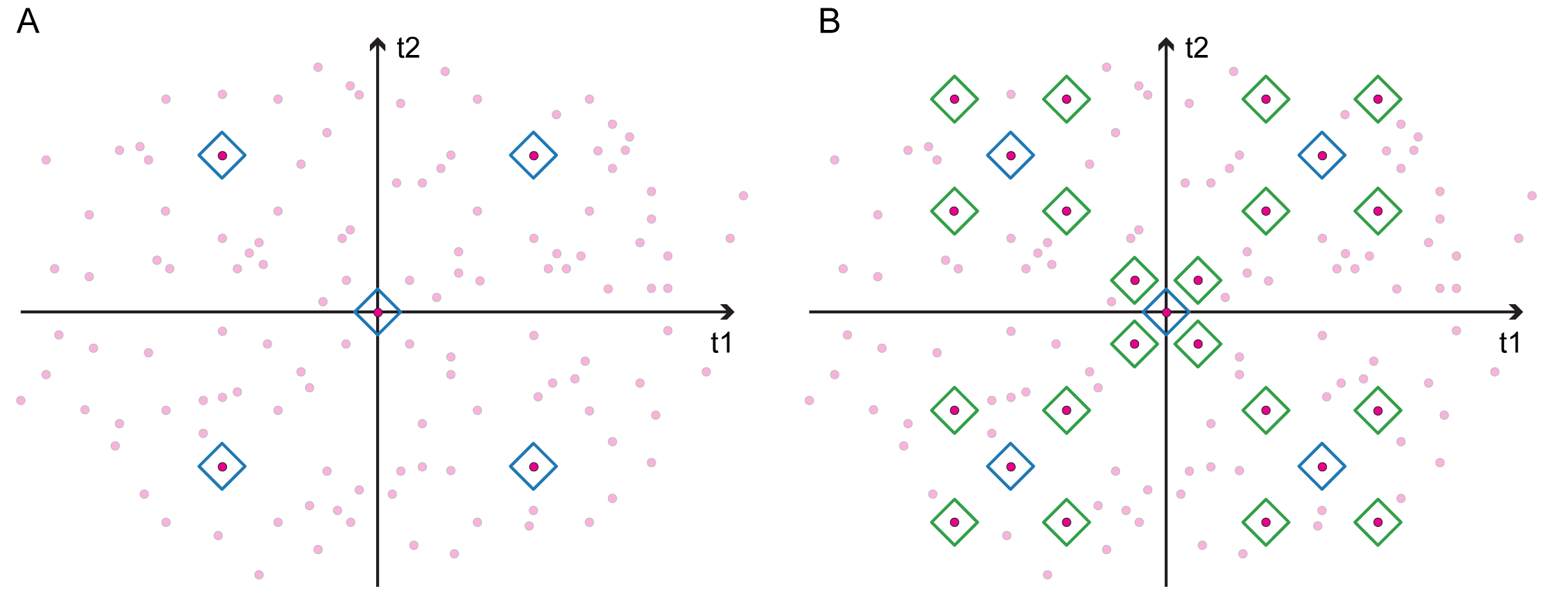

Supplement: S1 Fig — The figure illustrates a schematic score plot of the first and second component from a PCA model. Pink dots represent the data points (samples) and diamonds indicate selected samples. The baseline selection for all municipalities was made according to a full factorial design (A), whereas for the more populous municipalities an extended design was used (B). (TIF) [file pone.0213350.s002.tif]
